# Supplementary material for: A practical guide and power analysis for GLMMs: detecting among treatment variation in random effects
Source: PeerJ. 2015 Sep 17;3:e1226. doi: 10.7717/peerj.1226 (PMC4579019; doi:10.7717/peerj.1226)
Supplement: Supplemental Information 1 [file peerj-03-1226-s001.pdf]

Supplementary tutorial for "A Practical Guide and Power Analysis for GLMMs: Detecting Among Treatment Variation in Random Effects" using R

Kain, Morgan, Ben M. Bolker, and Michael W. McCoy

Introduction

The goal of this document is to provide the R code necessary to conduct the power analyses described in the main paper and instructions on how to sculpt simulations for random intercepts and random slopes GLMM for your specific research question.

Here we present simulations for a GLMM object using newly developed simulation capabilities of the `lme4` package. We provide a worked example for simulating data and running a power analysis for detecting differences in variation (among-individual variation in reaction norm intercept) by treatment. For each of the other target reaction norm variance parameters we provide the `lme4` syntax required to simulate and analyze data for comparisons of that variance parameter.

This tutorial is broken into five main segments:

1) Worked example for among-individual variation in intercept (σ^2_{0k})
    a) Data simulation and power analysis for a specific sampling scheme and effect size
    b) Quick extraction and visualization of results
2) Parameterization for the other two variance comparisons
    a) Among-individual variation in slope (σ^2_{1k})
    b) Within-individual variation in intercept (σ^2_{vk})
3) Syntax for a three-treatment model
4) Example nested loop structure for full simulation
5) Supplemental Table 1: Full parameter values for all simulations presented in the main text

1) Worked example for among-individual variation in intercept (σ^2_{0k})

For this worked example we discuss a two-treatment experiment whose goal is to determine if the magnitude of among-individual variation differs by treatment. We describe an experiment with a binomially distributed response variable. An example of an experiment that fits these criteria is an experiment testing if individual freshwater snails behave more/less variably from one another when exposed to predator cue than when exposed to no predator cue. Freshwater snails respond to predation risk by pumpkinseed sunfish by hiding under debris. The proportion of time individuals are observed to be hiding underneath a tile shelter following exposure to cue from a sunfish is one possible binomial response variable.

To conduct these simulations and power analyses you will need a recent version of `lme4`.

1) Step one is to set up a data frame containing information about the experimental design (number of treatments, number of individuals per treatment, number of observations per individual): expand.grid() is useful. However, note that expand.grid() results in a fully balanced design. To allow for some variation in the number of observations per individual, some modification will be needed.
If you have any continuous predictors or covariates, then you need to figure out what the distribution of that is going to be: is it regular/linear, or Normally distributed, or uniform? To include observation-level random effects/overdispersion (within-individual variation), then add an obs variable to the data frame which is defined simply as factor(seq(nrow(your_data))).

For this worked example we only use a single value for each experimental design variable. Once you have a simulation/estimation procedure working for a particular set of variables, you can embed it in a large, nested for loop that explores the whole range of experimental design variables you are interested in (e.g. effect size, variance, number of blocks, samples per block, etc.). An example nested for loop is provided in section 4 of this supplement.

Each variable value stored below corresponds to a single treatment. For example rep_vec sets the number of individuals sampled in *each treatment*. In the two treatment model presented below TSS for experiment is (rep_vec)*(repeat_vec)*2 = 180

Number of individuals

```
> rep_vec <- 15
```

Number of repeated measures within each individual

```
> repeat_vec <- 6
```

Among-individual variation in treatment 1 – Hereby referred to as the homogeneous (low-variation) treatment

```
> theta_among.homog_vec <- 0.2
```

Among-individual variation in treatment 2 – Hereby referred to as the variable (high-variation) treatment

```
> theta_among.var_vec <- 0.4
```

Observation level variation/overdisperison (within-individual variation)

```
> theta_obs_vec <- 0.2
```

Set up data frame:

```
> expdat <- expand.grid(indiv = factor(seq(rep_vec)),
+      obs = seq(repeat_vec), ttt = c("homog", "var"))

> expdat <- transform(expdat, homog = as.numeric((ttt ==
+     "homog")), var = as.numeric((ttt == "var")))

> expdat$total_obs <- factor(seq(nrow(expdat)))
```

We generally find it most convenient to store everything in a large multi-dimensional array, with one dimension for each experimental design variable, a dimension for replicates, and as many dimensions as necessary for the information you want to save about each replicate. For example, if you will be considering 10 possible numbers of samples per block, 10 possible numbers of blocks, and 5 possible effect sizes, doing 1000 replicates for each combination, and you wanted to keep information about the mean and standard deviation of 3 different parameters, you would end up with a 10 x 10 x 10 x 5 x 1000 x 3 x 2 array (Note this is an array of 30 *million* elements, representing 5,000,000 simulation runs – it's easy to get carried away with this sort of experiment!). Make sure to give dimnames to the array, where each element in the list itself has a name. For example, the full array used in the simulation run for the power analyses results presented in the paper was as follows:

```
> power_sim <- array(NA,dim=c(length(theta_among.homog_vec),
+     length(theta_among.var_vec), length(theta_obs_vec),
+     length(rep_vec), length(repeat_vec),nsim, 9),
+     dimnames=list(theta_h=theta_among.homog_vec,
+     theta_var=theta_among.var_vec,
+     theta_obs=theta_obs_vec,num.indiv=rep_vec,
+     repeat.meas=repeat_vec, sim.count=seq(nsim),
+     var=c("est","stderr","zval","ttt.pval","obsvar",
+    "indivvar.homog","indivvar.var","devdiff","var.pval")))
```

Keeping the data in an array this way makes it easy to select and/or average across the slices you want; when you want to convert the data to long format for analysis or plotting with lattice or ggplot, just use reshape2::melt(). A brief example using melt() and ggplot to visualize results is provided in supplement section 1b.

2) Specify the parameters: "theta" - in the case of single variance terms, which is just the standard deviation of each random effect: e.g. theta=c(1, 0.5) (among-individual variation is 4x among-observation variance). "beta" is the fixed-effects parameters on the logit scale, in this case (intercept, treatment).

```
> nsim <- 20
> beta <- c(0.5, 0)
> theta <- c(theta_obs_vec, theta_among.var_vec,
+     theta_among.homog_vec)
> .progress <- "text"
> verbose <- TRUE
```

Set up a matrix of NA in case of failure in model fitting

```
> errmat <- matrix(NA,nrow=nsim,ncol=9)
```

3) Set up the formula corresponding to the model you want to fit: For this example we want to simulate and estimate a case where the among-individual variation in intercept differs by treatment. See the posts on r-sig-mixed-models by David Afshartous <http://thread.gmane.org/gmane.comp.lang.r.lme4.devel/214> for additional information on how this works. Essentially, you have to construct your own numeric dummy variables. Simulate on this basis: simulate(formula, newdata, parameters, family = binomial) returns a response vector. See ?simulate.merMod for an additional example.

```
> ss2 <-simulate(~ttt+(0+homog|indiv:ttt)+(0+var|indiv:ttt)+
+    (1|total_obs), nsim=nsim, family=binomial,
+    weights=rep(5,nrow(expdat)),
+    newdata=expdat, newparams=list(theta=theta2,beta=beta))

> expdat$resp <- ss2[[1]]
```

4) Run the glmer and extract p-values and other model output.

Model for heterogeneous among-individual variation by treatment:

```
> fitfun <- function(expdat,i,.progress="text",verbose=TRUE){
+    fit2 <- try(glmer(resp~ttt+(0+homog|indiv:ttt)+
+    (0+var|indiv:ttt)+(1|total_obs), family=binomial,
+    weights=rep(5,nrow(expdat)),
+    data=expdat),silent=TRUE)
+            if(is(fit2,"try-error")) return(errmat)
+    fit2B <-
try(update(fit2,.~ttt+(1|indiv:ttt)+(1|total_obs)),
+    silent=TRUE)
+           if (is(fit2B,"try-error")) return(errmat)
+
+    fits=laply(seq(nsim),function(i)
+    fitsim2(i,models=list(fit2,fit2B)),.progress=.progress)
+    return(fits)
+    }
```

Fitting function to compare models:

```
> fitsim2 <- function(i,models=list(fit2,fit2B)) {
+    r1 <- try(refit(models[[1]],ss2[[i]]),silent=TRUE)
+    if (is(r1,"try-error")) return(rep(NA,9))
+    r1B <- try(refit(models[[2]],ss2[[i]]),silent=TRUE)
+    ss <- try(summary(r1))
+    cc <- if (is(ss,"try-error")) rep(NA,4) else
+    coef(summary(r1))["tttvar",]
```

```
+    res <- c(cc,unlist(VarCorr(r1)))
+    if (is(r1B,"try-error")) return(c(res,rep(NA,2)))
+    aa <- anova(r1,r1B)[2,]
+    devdiff <- unlist(c(aa["Chisq"]))
+    var.pval <- unlist(c(aa["Pr(>Chisq)"]))
+    return(c(res,devdiff,var.pval))
+    }
```

Run the power analysis and save the results. Here our power_sim array only has a single dimension for each experimental design variable.

```
> power_sim[1,1,1,1,1,,] <- fitfun(expdat, i = i)
```

Obtain power using reshape2::melt().

```
> d2 <- melt(power_sim, id.var = "var")
> d3 <- data.frame(d2[1:20, 1:6],
+    est = d2[d2$var=="est", ]$value,
+    stderr = d2[d2$var=="stderr", ]$value,
+    var.pval = d2[d2$var=="var.pval", ]$value)
> with(d3, mean(var.pval < 0.05))
```

Estimates:

```
> ggplot(arrange(d3, est), aes(x = seq(nsim), y = est,
+    ymin = est - 1.96 * stderr, ymax = est + 1.96 *
+    stderr)) + geom_pointrange() +
+    geom_hline(yintercept = mean(d3$est), colour = "red") +
+    theme_bw()
```

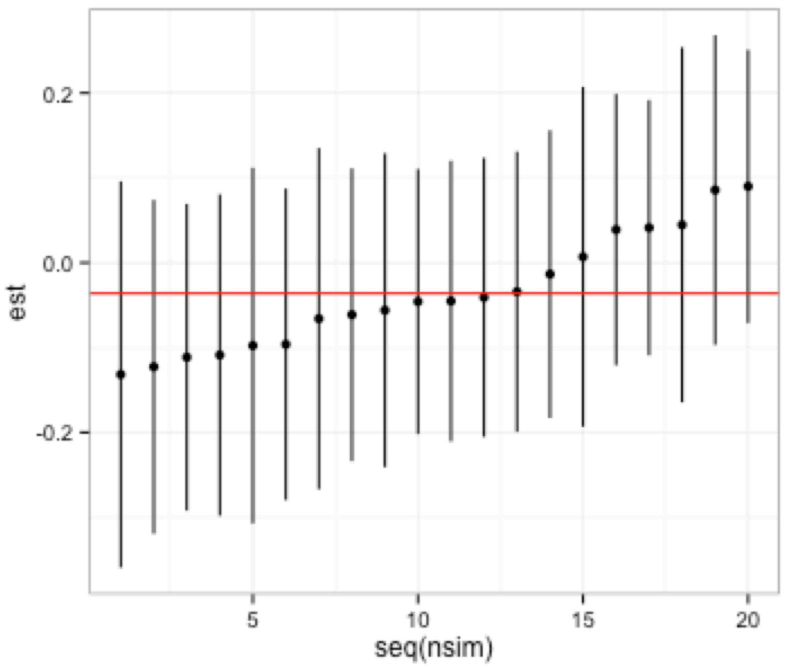

```
> truevals <- data.frame(variable = c("indivvar.homog",
+     "indivvar.var"), trueval = c(mean(d3$indivvar.homog),
+     mean(d3$indivvar.var)))

> d4 <- d2[d2$var=="indivvar.homog" | d2$var=="indivvar.var", ]

> ggplot(d4, aes(x = sim.count, y = value, colour = var)) +
+     geom_point(lwd = 3) + geom_hline(data = truevals,
+     aes(yintercept = trueval, colour = variable), lty = 2) +
+     theme_bw()
```

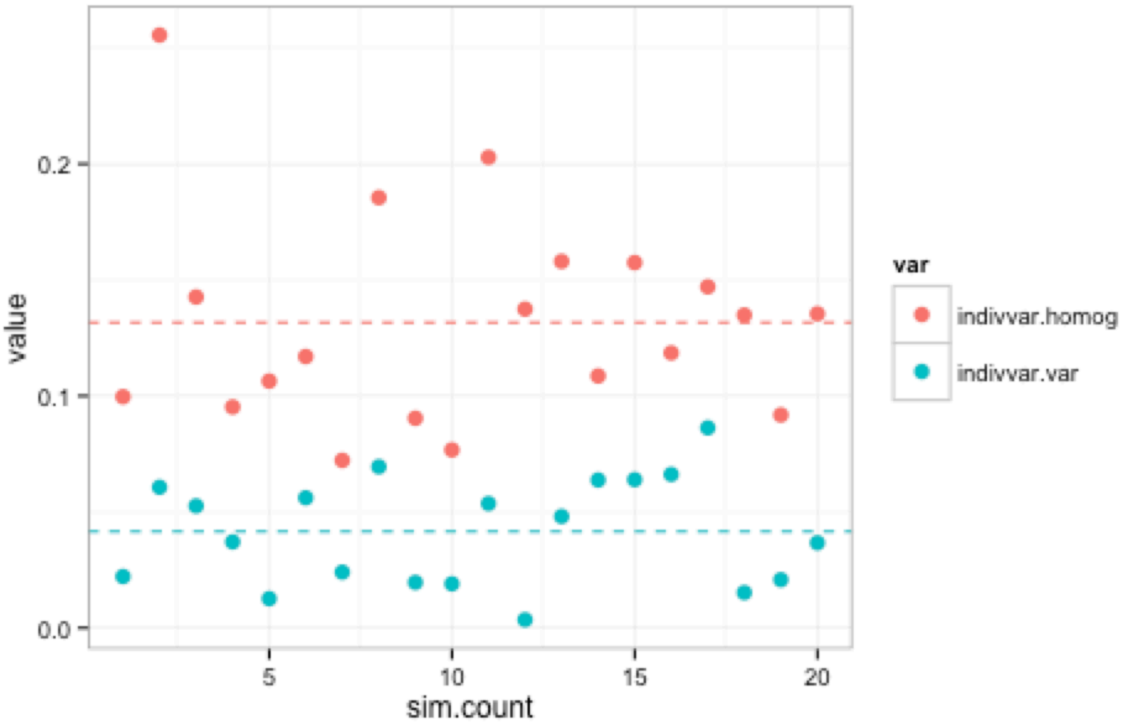

a) Among-Individual Variation in Slope

To model among-individual variation in slope the expand.grid() statement must be modified to treat obs as a sequential measurement.

```
> expdat <- expand.grid(indiv=factor(seq(rep_vec[i])),
+     obs=seq(repeat_vec[j]), ttt=c("homog","var"))

> expdat <- transform(expdat,
+     homog=as.numeric((ttt=="homog")),
+     var=as.numeric((ttt=="var")))

> expdat$total_obs <- factor(seq(nrow(expdat)))

> expdat <- transform(expdat, homogobs = homog*obs, varobs =
+     var*obs)
```

glmer code to model among-individual variation in slope:

```
> fit2 <- try(glmer(resp~ttt+(0+homogobs|indiv:ttt)+
+    (0+varobs|indiv:ttt)+(1|total_obs),
+    weights=rep(5,nrow(expdat)),
+    family=binomial,
+    data=expdat),silent=TRUE)

> fit2B <- try(update(fit2,.~ttt+(0+obs|indiv:ttt)+
+    (1|total_obs)),silent=TRUE)
```

b) Within-Individual Variation in Intercept

To model within-individual variation in intercept a new parameter is needed for the number of Bernoulli observations per sampling occasion (weight)

```
> fit2 <-try(glmer(resp~ttt+(1|indiv)+(0+homog|total_obs)+
+    (0+var|total_obs),family=binomial,weights=rep(weight[j],
+    nrow(expdat)),data=expdat),silent=TRUE)

> fit2B <- try(update(fit2,.~ttt+(1|indiv)+
+    (1|total_obs)),silent=TRUE)
```

3) Syntax for a three-treatment model

We will call our three treatments for the three-treatment model low, mid, and high

expand.grid() statement for a three treatment model:

```
> expdat <- expand.grid(indiv = factor(seq(rep_vec)),
+     obs = seq(repeat_vec), ttt = c("low", "mid", “high”))

> expdat <- transform(expdat,
+     low = as.numeric((ttt == "low")),
+     mid = as.numeric((ttt == "mid")),
+     high = as.numeric((ttt == “high)))

> expdat$total_obs <- factor(seq(nrow(expdat)))
```

lme4 syntax can be manipulated to provide estimates for any combination of treatments for any or all of the random effects included in the model. For a three treatment model for the random-intercept model presented previously, unique random effects can be estimates for each treatment for either among-individual or within-individual variation or both.

```
> glmer(resp~ttt+(0+low|indiv:ttt)+
+    (0+med|indiv:ttt)+(0+high|indiv:ttt)+(1|total_obs),
```

```
+    family=binomial,
+    weights=rep(repeat_vec,nrow(expdat)),
+    data=expdat),silent=TRUE)
```

For a unique random effect estimate for a single treatment (here the high treatment) and a single random effect estimate for the other two treatments (low and med treatments), a new "dummy variable" column is needed (containing a 1 for both low and med treatments and a 0 for the high treatment). Here we call this new column low_med

```
> glmer(resp~ttt+(0+high|indiv:ttt)+
+    (0+low_med|indiv:ttt)+(1|total_obs),
+    family=binomial,
+    weights=rep(repeat_vec,nrow(expdat)),
+    data=expdat),silent=TRUE)
```

Note that under LRTs where the difference in degrees of freedom between models > 1, more complicated adjustments are needed to correct for the conservative nature of the LRT when parameters are at the boundary of conceivable space (See Zuur et al. 2009)

4) Example Nested Loop Structure for Full Simulation

```
> for (t1 in seq_along(theta_among.homog_vec)) {
+  for (t2 in seq_along(theta_among.var_vec)) {
+    for(t3 in seq_along(theta_among.obs_vec)){
+      for (i in seq_along(rep_vec)) {
+        for (j in seq_along(repeat_vec)){
+
+      if (verbose) cat(t1,"/",length(theta_among.homog_vec)," ",
+                       t2,"/",length(theta_among.var_vec)," ",
+                       t3,"/",length(theta_among.obs_vec)," ",
+                       i,"/",length(rep_vec)," ",
+                       j,"/",length(repeat_vec)," ",
+                       "\n",
+                       sep="")
+
+     expdat <- expand.grid(indiv=factor(seq(rep_vec[i])),
+                           obs=seq(repeat_vec[j]),
+                           ttt=c("homog","var"))
+
+     expdat <- transform(expdat,
+                           homog=as.numeric((ttt=="homog")),
+                           var=as.numeric((ttt=="var")))
+     expdat$total_obs <- factor(seq(nrow(expdat)))
+
+     theta2 <- c(theta_among.obs_vec[t3],
+                           theta_among.var_vec[t2],
+                           theta_among.homog_vec[t1])
+
```

```
+     ss2 <- simulate(~ttt+(0+homog|indiv:ttt)+(0+var|indiv:ttt)+
+                         (1|total_obs),
+                         nsim=nsim,
+                         family=binomial,
+                         weights=rep(5, nrow(expdat)),
+                         newdata=expdat,
+                         newparams=list(theta=theta2,
+                         beta=beta))
+
+             expdat$resp <- ss2[[1]]
+
+             power_sim[t1,t2,t3,i,j,,] <- fitfun(expdat, i = i)
+
+         }
+       }
+     }
+   }
+ }
+    path <-"your_path.Rdata"
+    save("power_sim", file=path)
```

5) Table S1

Table S1: Full parameter values for all simulations. For example, Scenario 1: Figure 2C illustrates power to detect differences in σ^2_{0k} across ratios of individuals to sampling occasions with a TSS_T of 2,400 at effect sizes of 2x, 2.5x, and 3x difference in standard deviation by treatment.

**Target Variance**	**σ^2_{0k}**					**σ^2_{1k}**					**σ^2_{vk}**				
**Scenario**	**1**				**2**	**1**				**2**	**1**				**2**
**Figure**	**2A**	**2B**	**2C**	**5A**	**6A**	**3A**	**3B**	**3C**	**5B**	**6B**	**4A**	**4B**	**4C**	**5C**	**6C**
**Parameter**	Sampling Occasions			Bernoulli Obs	σ^2_{vk}	Sampling Occasions			Bernoulli Obs	σ^2_{vk}	Bernoulli Observations			Sampling Occasions	σ^2_{0k}
**TSS_T**	600	1,200	2,400	240 - 3,600	2,400	300	600	1,200	120- 1,800	1,200	600	1,200	2,400	240 - 3,600	2,400
**# Individuals**	2-60	2-120	2-240	120-2	2-240	2-30	2-60	2-120	60-2	2-120	2-60	2-120	2-240	2-120	2-240
**# Sampling Occasions**	60-2	120-2	240-2	2-120	240-2	30-2	60-2	120-2	2-60	120-2	5	5	5	1-15	5
**# Bernoulli Observations**	5	5	5	1- 15	5	5	5	5	1-15	5	60-2	120-2	240-2	120-2	240-2
**Effect Sizes**	2; 2.5; 3	2; 2.5; 3	2; 2.5; 3	2.5	2.5	2; 2.5; 3	2; 2.5; 3	2; 2.5; 3	2.5	2.5	2; 2.5; 3	2; 2.5; 3	2; 2.5; 3	2.5	2.5
**β_0**	0.5	0.5	0.5	0.5	0.5	0.5	0.5	0.5	0.5	0.5	0.5	0.5	0.5	0.5	0.5
**β_1**	-	-	-	-	-	0	0	0	0	0	-	-	-	-	-
**σ^2_{0homog}**	0.2	0.2	0.2	0.2	0.2	-	-	-	-	-	0.1	0.1	0.1	0.1	0.1-2
**σ^2_{0var}**	0.4, 0.5, 0.6	0.4, 0.5, 0.6	0.4, 0.5, 0.6	0.5	0.5	-	-	-	-	-	0.1	0.1	0.1	0.1	0.1-2
**σ^2_{1homog}**	-	-	-	-	-	0.2	0.2	0.2	0.2	0.2	-	-	-	-	-
**σ^2_{1var}**	-	-	-	-	-	0.4, 0.5, 0.6	0.4, 0.5, 0.6	0.4, 0.5, 0.6	0.5	0.5	-	-	-	-	-
**σ^2_{vhomog}**	0.1	0.1	0.1	0.1	0.1-2	0.1	0.1	0.1	0.1	0.1-2	0.2	0.2	0.2	0.2	0.2
**σ^2_{vvar}**	0.1	0.1	0.1	0.1	0.1-2	0.1	0.1	0.1	0.1	0.1-2	0.4, 0.5, 0.6	0.4, 0.5, 0.6	0.4, 0.5, 0.6	0.5	0.5
